# Trial to assess the tolerability of using felodipine to upregulate autophagy as a treatment of Huntington’s disease (FELL-HD): a phase II, single-centre, open-label, dose-finding trial protocol

**DOI:** 10.1136/bmjopen-2024-087983

**Published:** 2024-08-21

**Authors:** Katie Andresen, Emma Cutting, Dimitrios Apostolopoulos, Amy H Evans, Laura Oakley, Alimu Dayimu, Nikolaos Demiris, Katherine Bongaerts, Robyn Staples, Wendy Gooding, David C Rubinsztein, Roger A Barker

**Affiliations:** 1 Department of Clinical Neurosciences, University of Cambridge, Cambridge, UK; 2 Cambridge Clinical Trials Unit, Cambridge, UK; 3 Department of Pharmacy, Cambridge University Hospitals NHS Foundation Trust, Cambridge, UK; 4 Department of Medical Genetics, Cambridge Institute for Medical Research, The Keith Peters Building, Cambridge Biomedical Campus, Hills Road, Cambridge, UK; 5 UK Dementia Research Institute, University of Cambridge, Cambridge Institute for Medical Research, The Keith Peters Building, Cambridge Biomedical Campus, Hills Road, Cambridge, UK

**Keywords:** clinical trial, neurology, clinical trials

## Abstract

**Introduction:**

Huntington’s disease (HD) is an autosomal dominant neurodegenerative disorder that presents with a progressive movement disorder along with cognitive and psychiatric problems. It is caused by a Cytosine-adenin-guanine (CAG) expansion in exon 1 of the huntingtin gene which codes for mutant huntingtin (mHTT) that over time accumulates in cells, causing dysfunction and then death through new toxic gain-of-function mechanisms. Autophagy has been shown to be critical for the degradation of diverse intracytoplasmic aggregate-prone proteins that cause neurodegenerative disease, including mHTT. From a screen of a library enriched in approved drugs, felodipine was selected as the most suitable candidate showing strong autophagy-inducing effects in preclinical models of HD. We are, therefore, conducting a trial to assess the safety and tolerability of felodipine in people with early HD.

**Methods and analysis:**

FELL-HD is a phase II, single-centre, open-label, dose-finding trial in people with early HD. 18 participants with early clinical features of the disease will be treated with felodipine for 58 weeks, with a further 4-week follow-up. The primary outcome measure is the number of adverse events attributable to felodipine. Exploratory outcomes include additional measures of motor and cognitive function, non-motor symptoms and quality of life scales, as well as peripheral and central disease biomarkers assessed through brain MRI. Analysis of blood and cerebrospinal fluid will also be performed through an associated sample study, FELL HD-s.

**Ethics and dissemination:**

The study was approved by the London-Brent Research Ethics Committee (reference 22/LO/0387) and has been accepted by the Medicines and Healthcare products Regulatory Agency for clinical trials authorisation (reference CTA 12854/0256/001-0001). A lay summary of the results of the trial will be uploaded to our research group website which is publicly accessible. A webinar or in-person open day, to present results of the trial to participants and our wider cohort of patients who attend our centre, will be held once the trial is completed. The results of the trial will also be published in scientific journals and presented at national and international conferences.

**Trial registration numbers:**

EudraCT-2021-000897-27, ISRCTN56240656.

**STRENGTHS AND LIMITATIONS OF THIS STUDY**

- A comprehensive approach to investigate whether a drug repurposed to slow disease progression works through upregulating autophagy is feasible and well tolerated in patients with a progressive neurodegenerative disorder.
- The duration of treatment to study this with exploratory measures around efficacy which is possible given the trial is a yearlong.
- The open-label nature of the study may mean participants are biased to the effects of the medication and the small number of participants treated.
- We are unable to directly measure target engagement around autophagy as we have no biological measures to look at this process in the brain.

## Introduction

 Huntington’s disease (HD) is a progressive, genetic, neurodegenerative disorder that affects approximately 5700 people in the UK,^1^ with 5 times as many at risk of having inherited the gene. Clinical features typically emerge at the height of normal adult working life, namely between 30 and 50 years and include motor dysfunction, neuropsychiatric disturbances and cognitive difficulties. The disease runs a progressive course over about 20 years to death. There are currently only limited symptomatic therapies for HD, and these largely target only the motor features, there is, therefore, an urgent need for disease-modifying agents for people with HD.

The major pathological problem in HD relates to the production of abnormal huntingtin, called mutant huntingtin (mHTT), which over time aggregates and causes neuronal cells to dysfunction and then die. This production occurs as a result of inheriting the abnormal HD gene. Like many intracytoplasmic, aggregate-prone, mutant proteins linked to neurodegenerative diseases, the mHTT causes pathology via new toxic gain-of-function mechanisms. Given all this, it is clear that the logical way to try and stop HD is either to prevent the mHTT being formed (which is being investigated in trials using, eg, antisense oligonucleotides^2^) or clear it more efficiently from the cell.

One normal intracellular pathway that clears this and many abnormal proteins is called autophagy, which is initiated by double-membraned structures, called autophagosomes, that engulf portions of cytoplasm. Autophagosomes then end up fusing with lysosomes, where their contents are degraded.^3^ Autophagy has been shown to be critical for the degradation of diverse intracytoplasmic aggregate-prone proteins that cause neurodegenerative disease, including mHTT, as well as other proteins linked to neurodegenerative disorders of the brain^4^,^7^ and autophagy-upregulating drugs have been shown to enhance the clearance of these proteins, thereby attenuating their toxicities in Drosophila, zebrafish and mice (reviewed in Menzies *et al*^3^). This concept that autophagy upregulation may have therapeutic value in diverse diseases caused by aggregate-prone intracytoplasmic proteins has been subsequently validated by many groups and is now a major therapeutic target in neurodegeneration.

In preclinical studies, using a screen of a library enriched in approved drugs that induce autophagy, verapamil, an L-type calcium channel blocker, was identified as an inducer of autophagosome formation. Verapamil also reduced the percentage of cells with aggregates of mHTT in autophagy competent but not in autophagy-null cells.^8^ L-type calcium channel blockers are antihypertensive drugs and are widely used in the population so this family of drugs was considered to be suitable for repurposing for long-term treatment. As it was found verapamil does not cross the blood–brain barrier, this would make it unsuitable for the chronic treatment of primary central nervous disorders. A panel of currently prescribed L-type calcium channel blockers was screened to identify a blood–brain barrier-penetrant member that showed strong autophagy-inducing effects as well as having a long half-life in humans.^9^ Felodipine was selected as the most suitable candidate for further assessments. Felodipine has been found to induce autophagy in neurons in vitro and ameliorate in vivo toxicities in relevant animal models.^9^ Pharmacokinetic studies have also been performed in mice to determine the optimal treatment regime, which would mimic the plasma concentration that the drug reaches in humans at currently prescribed doses of 10 mg/day (the standard dose used for treating hypertension). Subcutaneous minipumps were used in mice with a protocol that mimicked the plasma concentrations of the drug seen in humans taking felodipine, and good brain penetrance of the drug was found with some behavioural benefits.^9^ However, to truly verify this, longer studies would be needed but these could not be performed because of Home Office restrictions on the long-term use of osmotic minipumps in mice.

Further support for this approach comes from epidemiological studies, which suggest that the use of felodipine may reduce the incidence of another disease associated with protein aggregation, Parkinson’s disease, in hypertensive patients.^10^ Additionally, the safety profile of felodipine is already established and is licensed to treat hypertension. The average half-life of felodipine in the terminal phase is 25 hours, and steady state is reached after 5 days. There is no significant accumulation during long-term treatment and the medication only needs to be administered once a day. All of this, in combination with the preclinical work, supported the decision to test felodipine in people with early HD into a clinical trial.

## Methods and analysis

### Overview

FELL-HD is a phase II, open-label trial in people with early HD that aims to test the safety and tolerability of different doses of felodipine. The trial will investigate whether three different doses of felodipine are tolerated over a 58-week treatment period, followed by a 4-week washout follow-up period.

**Figure 1:**
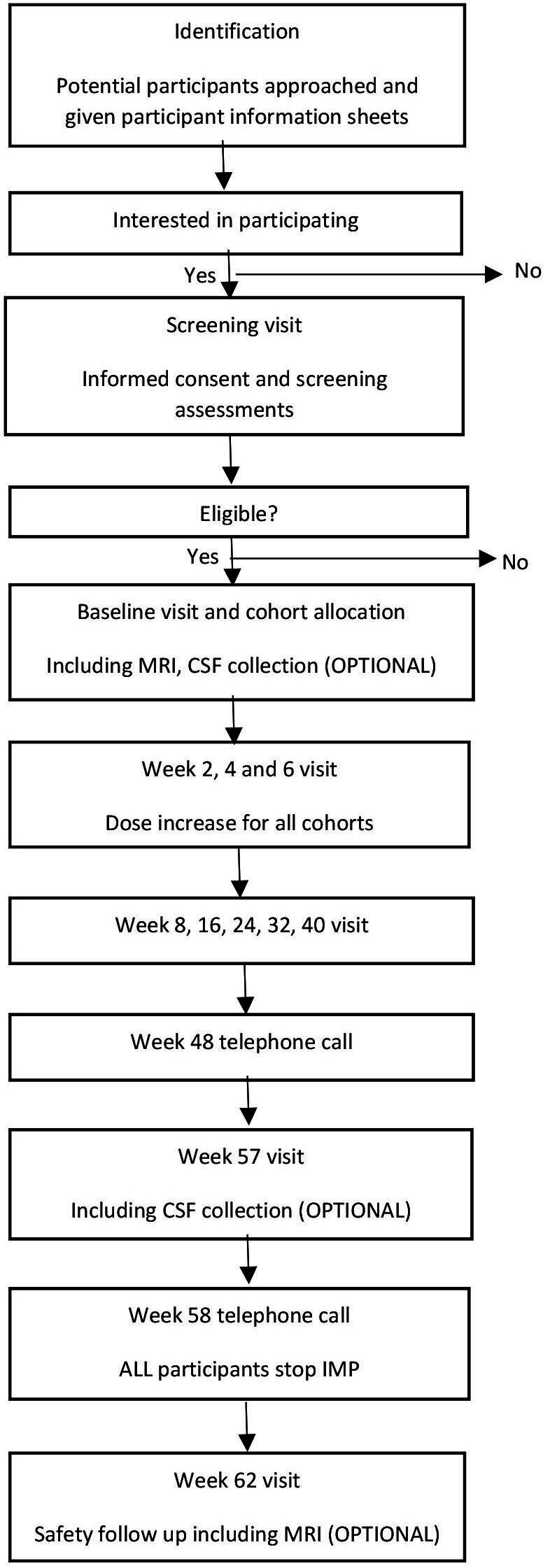
Flow chart. CSF, cerebrospinal fluid; IMP, Investigational medicinal product

18 participants will be recruited and allocated to one of three cohorts in alternating fashion that will all receive felodipine. All participants will begin on 2.5 mg of felodipine with the dose being increased every 2 weeks from baseline to the cohort maximum dose, only if the previous dose has been tolerated. Cohort 1 will receive a maximum of 5 mg, cohort 2 will receive a maximum of 10 mg and cohort 3 will receive a maximum of 20 mg. The dosing schedule is summarised in table 1. Safety assessments will be performed every 2 weeks up to week 8 while participants reach their maximum allocated dose. Following this, clinical and safety assessments will be performed every 8 weeks until week 40. At week 48, a telephone visit will be performed and participants will then be seen at week 57 for safety and clinical assessments. A repeat telephone visit will be performed at week 58 which is when participants will end their treatment phase with felodipine. Clinical and safety assessments will then be performed at week 62 for the final trial visit. For the schedule of visits, refer to figure 1.

**Table 1:** Dosing schedule for all cohorts

Time point	Felodipine dose (mg)		
	Cohort 1	Cohort 2	Cohort 3
Week 0	2.5	2.5	2.5
Week 1	2.5	2.5	2.5
Week 2	5	5	5
Week 3	5	5	5
Week 4	5	10	10
Week 5	5	10	10
Week 6–57	5	10	20
Week 58	Stop	Stop	Stop

All participants involved in the FELL-HD trial will also be asked if they would like to participate in an associated sample study called FELL-HD-s (Full title: A trial to assess markers of autophagy in patients with Huntington’s disease who have been treated with felodipine, IRAS 297596). The FELL-HD-s study involves research blood samples being taken at the participants’ FELL-HD trial visits, as well as an optional component to collect cerebrospinal fluid (CSF) at baseline and week 57. The CSF collection is optional to ensure that this inclusion does not impact recruitment.

While the primary objective of the FELL-HD trial is safety, we will also look at changes in clinical measurements of HD using validated motor and cognitive assessments between baseline and week 62. Changes in volumetric measures on MRI will also be evaluated as an exploratory measure if participants consent to this, as having an MRI is an optional component of the trial. In addition, our FELL-HD-s study will assess markers of target engagement of felodipine in blood and CSF samples.

### Patient and public involvement

People with HD who attend our National Health Service (NHS) HD clinic at the John Van Geest Centre for Brain Repair (VGB), University of Cambridge, gave input into the trial design. A patient and public involvement (PPI) panel of four patients who have been/are actively involved in other research projects through the clinic, provided feedback on the trial design and the commitment required by the participants. The PPI panel also reviewed our participant information sheets for clarity to ensure they were detailed enough and easy to understand, and a member of the panel attended the ethical committee meeting.

### Participant identification

**Figure 2:**
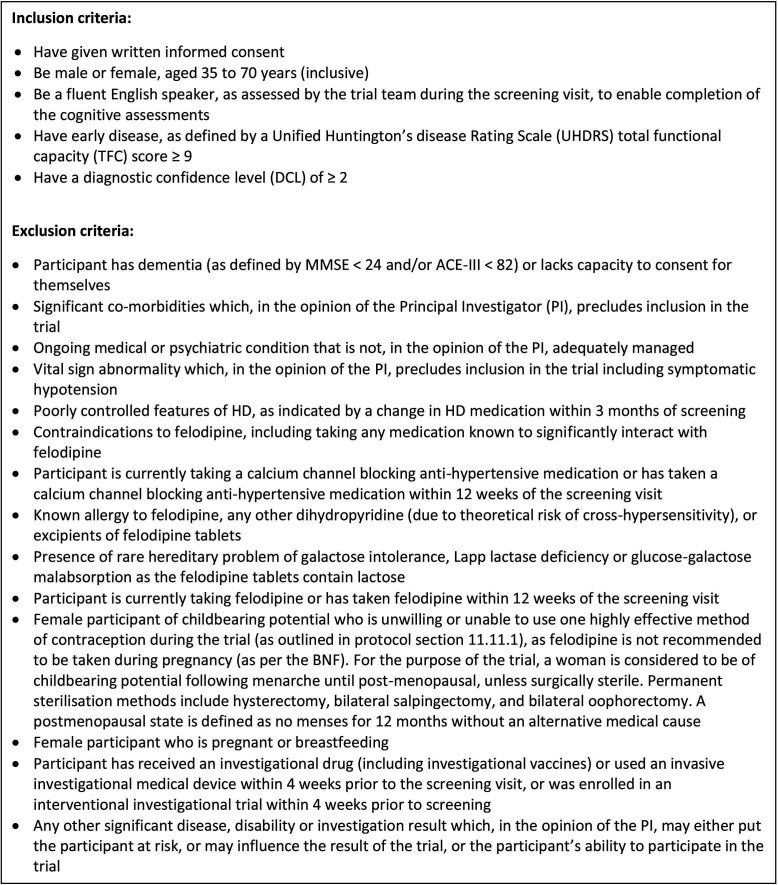
Inclusion/exclusion criteria. ACE-III, Addenbrookes Cognitive Examination III; HD, Huntington’s disease; MMSE, Mini Mental State Examination.

Participants will be recruited from a single site in Cambridge, UK. Potential participants will be identified via the NHS HD clinic and from the database of people who have previously attended the NHS HD clinic. These will only be people who have indicated they are interested in being contacted about involvement in future research. The database holds detailed information about demographics, HD status and clinical assessments, comorbidities and medication that will assist in identifying potential participants that fit the inclusion/exclusion criteria as outlined in figure 2.

Alternatively, participants may be asked if they would be interested in the trial at their routine NHS HD clinic appointment.

All interested participants will be provided with a participant information sheet and after approximately 2 weeks (to give them sufficient time to consider the invitation), the trial team will contact them by telephone to see if they would like to participate. If the potential participant is interested, they will be invited to a screening visit where the participant’s understanding of the trial is confirmed and written informed consent is obtained before confirming eligibility through screening assessments. Informed consent will be taken by the principal investigator (PI) or subinvestigator (SI). However, if a potential participant is not interested, they will continue with their standard clinical care.

### Eligibility criteria

A potential participant will be considered eligible for recruitment to the FELL-HD trial if they meet the inclusion/exclusion criteria listed in figure 2. At the screening visit, a review of the participant’s medical history, demographics, vital signs, physical examination, safety blood sampling and clinical assessments will be used to determine eligibility.

Any participants who are deemed eligible to participate in the FELL-HD trial will be assessed for eligibility to take part in the sample study FELL-HD-s if they consent to this. Participants who consent to FELL-HD-s will be screened for HIV, hepatitis B virus (HBV) or hepatitis C virus (HCV) to determine eligibility.

### Outcome measures

The primary outcome measure for the FELL-HD trial is the number of adverse events (AEs) attributable to felodipine. An AE is any untoward medical occurrence in a participant that may or may not have a causal relationship to the treatment. Recording of AEs starts from the point of informed consent at the screening visit regardless of whether a participant has started the treatment or not.

Exploratory outcome measures of the trial include:

1. Changes in clinical measurements of HD using validated motor and cognitive assessment tools between baseline visit (week 0) and end of the trial (week 62).
2. Change on a range of MRI volumetric measures between baseline (week 0) and end of the trial (week 62).

In the sample-study FELL-HD-s, the primary outcome measure is the change in mHTT levels in the blood between baseline (week 0) and end of the trial (week 62). The secondary outcome measurement is the change in neurofilament light chain (NFL) levels in the blood between baseline (week 0) and end of the trial (week 62).

Exploratory outcome measures of the FELL-HD-s-study include:

1. Change in level of felodipine in the blood between baseline and week 62.
2. Change in level of felodipine in the CSF between baseline and week 57.
3. Change in mHTT levels in the CSF between baseline and week 57.
4. Change in NfL levels in the CSF between baseline and week 57.
5. Change in level of glial fibrillar acidic protein (GFAP) in the blood between baseline and week 62.
6. Change in level of GFAP in CSF between baseline and week 57.

### Sample size calculation

As this is the first trial to investigate felodipine in patients with HD, there are insufficient data to conduct a formal assessment of the anticipated effect size. The preliminary data from this trial will provide the necessary information to design a further phase 3 trial that is sufficiently powered to show this.

### Trial procedures

#### Clinical

All clinical visits will take place at the John Van Geest Centre for Brain Repair in Cambridge, UK. Clinical measures assessing motor and non-motor features of HD will be performed at screening, baseline and every 8 weeks thereafter up to week 40. After this time point, these clinical assessments will then be repeated at week 57 and at the end of the trial at week 62. The schedule of assessments is summarised in online supplemental table 2.

The Unified Huntington’s Disease Rating Scale (UHDRS) is a clinical rating scale used to assess four domains of clinical performance and capacity in HD; motor and cognitive function, behavioural abnormalities and functional capacity. The assessment has been validated and appears to be appropriate for repeated administration during clinical studies.^11^ Assessments include total functional capacity, diagnostic confidence level, total motor score (TMS), functional assessment and a battery of cognitive tests. Where participants consent, the UHDRS TMS section only may be video recorded at baseline and week 62, enabling validation of the scoring by an independent assessor to check inter-rater reliability.

Cognition will be assessed using the Addenbrookes Cognitive Examination III (ACE-III) which is a neuropsychological test used to identify cognitive impairment. The ACE-III provides a global measure of cognition as well as subscores in five domains: attention and orientation, memory, fluency, language and visuospatial function.^12^ This test will be used throughout the trial but at the screening visit, it will be used to confirm the participant’s capacity to consent.

Other non-motor aspects of HD will be evaluated using the Problem Behaviours Assessment-short), which is a semistructured interview used to rate behavioural symptoms of HD. The assessment looks at depression, anxiety, irritability, aggression, apathy, perseverative thinking, obsessive–compulsive behaviours, delusions, hallucinations, disorientation and will be administered by a trained member of the trial team.^13^ We will also use the Columbia Suicide Severity Rating Scale to rate if there is any suicidal ideation and behaviour on a scale ranging from ‘wish to be dead’ to ‘active suicidal ideation with a specific plan and intent’, participants will answer yes or no to the questions and any that have an answer of yes, more details will be captured. This assessment will be administered by a trained member of the trial team.^14^ Finally, the Hospital Anxiety and Depression Scale will be used which is a self-rating scale to measure anxiety and depression.^15^

Vital signs will be assessed closely, in particular, measurement of blood pressure due to felodipine being an antihypertensive drug. Vital signs (blood pressure, pulse, respiratory rate and body temperature) will be measured at all clinic visits. Participants will also be asked to take weekly blood pressure readings at home and complete a diary that will be reviewed at all clinic visits by the trial team. Participants will be required to inform the trial team if blood pressure readings drop below 90/60 mm Hg or if they experience symptoms of low blood pressure, where further monitoring may be required as decided by the trial team.

Participants’ concomitant medications will be recorded while they are on the trial (including any dose changes), and participants may continue to take all regular medications as prescribed. Participants on any medications that contraindicate the use of felodipine will be excluded from participating in the trial.

#### MRI

The MRI will be an optional component of the trial and, if participants consent to this, an MRI will be performed at baseline (week 0) and at the end of the trial (week 62). Scanning will be conducted on a 3T MRI scanner at the Wolfson Brain Imaging Centre in Cambridge. Each scan will take approximately 1 hour. The scans will be used to assess whether there are any changes in the volume of the caudate nucleus, putamen and whole brain as well as assessing white matter integrity. Given that some participants may struggle to complete the MRI, if they consent to this procedure but would like some sedation, this will be offered to the participant.

#### Biosample collection and processing

As part of the FELL-HD trial, full blood count, liver function tests and urea and electrolytes samples will be taken at every clinic visit and analysed at the Laboratory Research Services in Addenbrooke’s Hospital.

If participants also consent to the FELL-HD-s substudy, 7.5 mL of blood will be collected and tested for HIV, HBV and HCV status to confirm eligibility. Starting at the baseline (week 0) visit and every 8 weeks up to week 40 and then at week 57 and week 62, up to 75 mL of blood will be collected for analysis of markers of disease, as well as levels of felodipine in the blood.

Samples will be taken in two different types of blood collection tubes depending on which laboratories the specimens will be sent to. Two samples will be taken in 8 mL sodium heparin cell preparation tubes and centrifuged at centrifugal force (RCF) 1500×g for 30 min at room temperature for extraction of plasma. Three samples will be taken in 6 mL K2E blood collection tubes and centrifuged at RCF 1300×g for 10 min at 4°C for extraction of plasma.

If consented, CSF will be collected via lumbar puncture at baseline (week 0) before treatment commences and week 57, 1 week prior to the end of treatment. The CSF will be used to assess levels of felodipine, NfL, mHTT and GFAP. CSF will be centrifuged at RCF 400×g for 10 min at 4°C. After spinning, the CSF supernatant will be separated from the CSF cells and stored.

All aliquots for blood and CSF will be stored at −80°C until the end of the trial where they will then be sent to the analysing laboratory which will look at the above protein levels.

### Treatment allocation and dosing

Participants will be assigned to one of three dosing cohorts. Cohort 1 will receive a total of 5 mg daily, cohort 2 will receive a total of 10 mg daily and cohort 3 will receive a total of 20 mg daily. There will be six participants assigned to each cohort in an alternating fashion, that is, participant 1 will be allocated to cohort 1, participant 2 will be allocated to cohort 2, participant 3 will be allocated to cohort 3, participant 4 will be allocated to cohort 1 and so on. This will be performed by the trial coordinator.

All participants will start on 2.5 mg from baseline (week 0), if tolerated, all participants will have their dose increased to 5 mg at week 2 and cohort 1 will then remain on 5 mg until week 58. Those assigned to cohort 2 will have their dose, if tolerated, increased again to 10 mg at week 4 and will remain on 10 mg until week 58. Those assigned to cohort 3 will also have their dose increased to 10 mg at week 4 and then have their dose, if tolerated, increased for a third time to 20 mg at week 6 and will remain on 20 mg until week 58. Please refer to the dosing schedule in table 1.

The dose will only be increased where the previous dose has been tolerated, all judgements of intolerance will be at the PI discretion. Intolerance may be defined by an occurrence of an AE of moderate severity, which is either definitely or probably related to the felodipine. If a participant is unable to tolerate a dose, they will be moved back to the last dose they were able to tolerate within their cohort dosing. If the participant is already at the lowest dose then felodipine will be withdrawn. No dose rechallenge will be undertaken if a dose is reduced during the course of the trial.

### Trial treatment withdrawal

Participants have the right to withdraw from the trial or trial treatment at any time, without giving a reason. Participants who withdraw from the trial or treatment will be given options in relation to future participation in the trial. They will be able to:

- Withdraw from trial treatment only, but continue with all safety and efficacy assessments on the trial.
- Undergo a final safety close-out visit prior to full withdrawal, see online supplemental table 1.
- Withdraw from trial treatment and follow-up visits, no further tests will be performed and no further data or samples will be collected.

### Trial monitoring and oversight

FELL-HD is jointly sponsored by Cambridge University Hospitals NHS Foundation Trust and University of Cambridge. The Sponsor will assign a Clinical Trials Monitor who will monitor the trial team to ensure they are adhering to the protocol and regulatory requirements. The first monitoring visit will take place within 10 working days of the first participant commencing treatment. Monitoring will then take place regularly until the trial ends. Any changes to the monitoring plan will be updated in the monitoring plan agreement and risk assessment form associated with the FELL-HD trial.

There will be a Trial Management Group (TMG) consisting of the chief investigator, SI, trial coordinators, trial pharmacist, trial statistician and clinical trial monitor who will meet once a month to discuss the running of the trial.

There will also be a Trial Steering Committee (TSC), which is a multidisciplinary group comprising members who are jointly responsible for the overall supervision of the FELL-HD trial. This committee will include but is not limited to the members of the TMG, an independent chair, independent clinical or scientific experts, independent statisticians with relevant experience and representatives of people with lived experience of HD. The TSC will meet at least every 12 months while participants are receiving felodipine and at least once thereafter.

All trial documentation and any amendments will be reviewed by the sponsor and approved for submission to the relevant regulatory bodies. Once full approval has been received, the information will be communicated as appropriate with the trial team, TMG and TSC.

All personal data are stored on the University of Cambridge Secure Data Hosting Service, this is password protected and can only be accessed by the immediate trial team. All data entered into the electronic database are anonymised and entered under a specific participant trial ID for the wider trial team to access.

### Statistical methods

A statistical analysis plan (SAP) will be finalised and approved before the commencement of the analysis. The analysis will be performed on the modified intention-to-treat population, which includes all eligible participants who had at least one dose of investigational treatment and were not replaced during the trial. Participant data will be analysed according to their allocated cohort. The primary outcome is felodipine-related AEs, therefore, missing data are not expected. In all analyses, no imputation will be performed.

Participant data will be summarised by cohort. For the primary endpoint, no formal hypothesis testing will be performed. The statistical analysis will be mostly descriptive, with the number of felodipine-related AEs summarised by dosage for each cohort. All the exploratory endpoints described in the outcome measurement section will be summarised, and a significance test will be performed between baseline and week 57/62, using a paired t-test as the primary analysis and a Wilcoxon signed-rank test as the sensitivity analysis. No formal subgroup analysis will be performed. This trial has no predetermined ending criteria or formal interim analysis.

## Ethics and dissemination

The FELL-HD trial was approved by the London-Brent Research Ethics Committee, reference 22/LO/0387 along with acceptance from the Medicines and Healthcare products Regulatory Agency, reference CTA 12854/0256/001-0001. The FELL-HD-s sample study was approved by the East Midlands-Nottingham 1 Research Ethics Committee, reference 22/EM/0007.

Results of the trial will be disseminated via our annual HD newsletter which participants of the trial can consent to receive during the consent process. A lay summary will also be uploaded to our research group website which is publicly accessible. We will hold a webinar or in-person open day, to present the final results of the trial to participants as well as our wider cohort of patients who attend our NHS HD clinic. The results of the trial will also be published in scientific journals and presented at national and international conferences.

Only fully deidentified data will be passed to the public domain, that is, on an open-access data repository/journal once sufficient validation has been conducted and meaningful analysis and publication are complete.

## supplementary material

10.1136/bmjopen-2024-087983 online supplemental file 1

## References

[1] Evans SJW, Douglas I, Rawlins MD (2013). Prevalence of adult Huntington’s disease in the UK based on diagnoses recorded in general practice records. *J Neurol Neurosurg Psychiatry*.

[2] Tabrizi SJ, Leavitt BR, Landwehrmeyer GB (2019). Targeting huntingtin expression in patients with huntington’s disease. N Engl J Med.

[3] Menzies FM, Fleming A, Caricasole A (2017). Autophagy and neurodegeneration: Pathogenic mechanisms and therapeutic opportunities. Neuron.

[4] Berger Z, Ravikumar B, Menzies FM (2006). Rapamycin alleviates toxicity of different aggregate-prone proteins. Hum Mol Genet.

[5] Ravikumar B, Duden R, Rubinsztein DC (2002). Aggregate-prone proteins with polyglutamine and polyalanine expansions are degraded by autophagy. Hum Mol Genet.

[6] Ravikumar B, Vacher C, Berger Z (2004). Inhibition of mTOR induces autophagy and reduces toxicity of polyglutamine expansions in fly and mouse models of Huntington disease. Nat Genet.

[7] Webb JL, Ravikumar B, Atkins J (2003). Alpha-Synuclein is degraded by both autophagy and the proteasome. J Biol Chem.

[8] Williams A, Sarkar S, Cuddon P (2008). Novel targets for Huntington’s disease in an mTOR-independent autophagy pathway. Nat Chem Biol.

[9] Siddiqi FH, Menzies FM, Lopez A (2019). Felodipine induces autophagy in mouse brains with pharmacokinetics amenable to repurposing. Nat Commun.

[10] Lee YC, Lin CH, Wu RM (2014). Antihypertensive agents and risk of Parkinson’s disease: a nationwide cohort study. PLoS One.

[11] Huntington Study Group (1996). Unified Huntington’s disease rating scale: Reliability and consistency. Mov Disord.

[12] Hsieh S, Schubert S, Hoon C (2013). Validation of the addenbrooke’s cognitive examination III in frontotemporal dementia and alzheimer’s disease. Dement Geriatr Cogn Disord.

[13] McNally G, Rickards H, Horton M (2015). Exploring the validity of the short version of the problem behaviours assessment (PBA-s) for huntington’s disease: A rasch analysis. J Huntingtons Dis.

[14] Posner K, Brown GK, Stanley B (2011). The columbia-suicide severity rating scale: initial validity and internal consistency findings from three multisite studies with adolescents and adults. Am J Psychiatry.

[15] Bjelland I, Dahl AA, Haug TT (2002). The validity of the hospital anxiety and depression scale. J Psychosom Res.

