# Supplementary material for: Trial to assess the tolerability of using felodipine to upregulate autophagy as a treatment of Huntington’s disease (FELL-HD): a phase II, single-centre, open-label, dose-finding trial protocol
Source: BMJ Open. 2024 Aug 21;14(8):e087983. doi: 10.1136/bmjopen-2024-087983 (PMC11340714; doi:10.1136/bmjopen-2024-087983)
Supplement: online supplemental file 1 [file bmjopen-14-8-s001.pdf]

Supplementary table 1

Visit time point	Screening (week -4 to day -1)	Baseline (week 0)	Week 2 (+/- 3 days)	Week 4 (+/- 3 days)	Week 6 (+/- 3 days)	Week 8 (+/- 3 days)	Week 16 (+/- 1 week)	Week 24 (+/- 1 week)	Week 32 (+/- 1 week)	Week 40 (+/- 1 week)	Week 48 (phone) (+/- 1 week)	Week 57 (+/- 1 week)	Week 58 (phone) (+/- 1 week)	Week 62 (+/- 1 week)	Safety follow-up (+/- 1 week)
**General assessments**															
Informed consent	X														
Medical history	X														
Demographics	X														
Physical examination	X														
Concomitant medication review	X	X	X	X	X	X	X	X	X	X	X	X	X	X	
Vital signs[1]	X	X	X	X	X	X	X	X	X	X		X		X	X
Blood pressure diary review			X	X	X	X	X	X	X	X	X[2]	X	X[2]	X	X
AE assessment	X	X	X	X	X	X	X	X	X	X	X	X	X	X	X
Calculation of CAP-S score	X														
**Biological samples**															
Pregnancy test	X	X		X		X	X	X	X	X		X		X	X
Blood sampling[2]	X	X	X	X	X	X	X	X	X	X		X		X	X
**Clinical assessments**															
MMSE	X														
UHDRS[3]	X	X				X	X	X	X	X		X		X	
ACE-III	X	X				X	X	X	X	X		X		X	
HADS	X	X				X	X	X	X	X		X		X	
PBA-s	X	X				X	X	X	X	X		X		X	
C-SSRS	X	X				X	X	X	X	X		X		X	
**Imaging**															
MRI (**optional**)		X												X	
**Trial treatment**															
Felodipine commencement		X													
Felodipine dose increase			All cohorts	Cohorts 2 and 3	Cohort 3 only										
Felodipine treatment cessation													All cohorts		
Felodipine dispensing		X	X	X	X	X	X	X	X	X					
Compliance check			X	X	X	X	X	X	X	X	X[8]	X		X	X

Supplementary table 1:

1 Following IMP commencement, participants will be instructed to perform weekly blood pressure readings at home using a blood pressure monitor provided by the trial team. Participants will not need to complete readings on weeks that they have an in-person trial visit.

2 Blood sampling for safety labs: FBC, LFTs, U&Es

Supplementary table 1

3 UHDRS will include TFC, DCL, TMS, functional assessment, Stroop colour naming, Stroop word reading, Stroop interference, SDMT and verbal fluency. This assessment may be recorded at the baseline visit, and at subsequent visits, if participants consent to this.
ACE-III, Addenbrooke's Cognitive Examination-III; AE, Adverse event; C-SSRS, Columbia Suicide Severity Rating-Scale; HADS, Hospital Anxiety and Depression Scale; MMSE, Mini Mental State Examination; MRI, Magneotic Resonance Imaging; PBA-s, Problem Behaviours Assessment-Short; UHDRS, Unified Huntington's Disease Rating Scale.
