# Supplementary material for: Trial to assess the tolerability of using felodipine to upregulate autophagy as a treatment of Huntington’s disease (FELL-HD): a phase II, single-centre, open-label, dose-finding trial protocol
Source: BMJ Open. 2024 Aug 21;14(8):e087983. doi: 10.1136/bmjopen-2024-087983 (PMC11340714; doi:10.1136/bmjopen-2024-087983)
Supplement: Supplementary file 2 [file bmjopen-2024-087983.reviewer_comments.pdf]

PEER REVIEW HISTORY

BMJ Open publishes all reviews undertaken for accepted manuscripts. Reviewers are asked to complete a checklist review form (**http://bmjopen.bmj.com/site/about/resources/checklist.pdf**) and are provided with free text boxes to elaborate on their assessment. These free text comments are reproduced below.

ARTICLE DETAILS

**TITLE (PROVISIONAL)**	A trial to assess the tolerability of using felodipine to upregulate autophagy as a treatment of Huntington's disease (FELL-HD): a Phase II, single centre, open label, dose finding trial protocol
**AUTHORS**	Andresen, Katie; Cutting, Emma; Apostolopoulos, Dimitrios; Evans, Amy H; Oakley, Laura; Dayimu, Alimu; Demiris, Nikolaos; Bongaerts, Katherine; Staples, Robyn; Gooding, Wendy; Rubinsztein, David; Barker, Roger A

This article was not externally reviewed at BMJ Open. Protocols that have been independently assessed prior to submission to BMJ Open are usually fast-tracked to publication on the grounds that further substantial changes will not be possible. This independent assessment will usually be external, independent review for both a substantial grant award from a non-commercial or government funder and ethics approval.
